# Gene Network Analysis in a Pediatric Cohort Identifies Novel Lung Function Genes

**DOI:** 10.1371/journal.pone.0072899

**Published:** 2013-09-02

**Authors:** Bruce A. Ong, Jin Li, Joseph M. McDonough, Zhi Wei, Cecilia Kim, Rosetta Chiavacci, Frank Mentch, Jason B. Caboot, Jonathan Spergel, Julian L. Allen, Patrick M. A. Sleiman, Hakon Hakonarson

**Affiliations:** 1 Division of Pulmonary Medicine and Cystic Fibrosis Center, The Children’s Hospital of Philadelphia, Philadelphia, Pennsylvania, United States of America; 2 Center for Applied Genomics, The Children’s Hospital of Philadelphia, Philadelphia, Pennsylvania, United States of America; 3 Department of Computer Science, New Jersey Institute of Technology, Newark, New Jersey, United States of America; 4 Division of Pediatric Pulmonology, Madigan Army Medical Center, Tacoma, Washington, United States of America; 5 Center for Pediatric Eosinophilic Disorders, The Children’s Hospital of Philadelphia, Philadelphia, Pennsylvania, United States of America; 6 Division of Human Genetics, The Children’s Hospital of Philadelphia, Philadelphia, Pennsylvania, United States of America; 7 Department of Pediatrics, University of Pennsylvania School of Medicine, Philadelphia, Pennsylvania, United States of America; 8 Division of Allergy and Immunology, The Children’s Hospital of Philadelphia, Philadelphia, Pennsylvania, United States of America; New Jersey Institute of Technology, United States of America

## Abstract

Lung function is a heritable trait and serves as an important clinical predictor of morbidity and mortality for pulmonary conditions in adults, however, despite its importance, no studies have focused on uncovering pediatric-specific loci influencing lung function. To identify novel genetic determinants of pediatric lung function, we conducted a genome-wide association study (GWAS) of four pulmonary function traits, including FVC, FEV_1_, FEV_1_/FVC and FEF_25–75%_ in 1556 children. Further, we carried out gene network analyses for each trait including all SNPs with a P-value of <1.0×10^−3^ from the individual GWAS. The GWAS identified SNPs with notable trends towards association with the pulmonary function measures, including the previously described *INTS12* locus association with FEV1 (p_meta_ = 1.41**×**10^−7^). The gene network analyses identified 34 networks of genes associated with pulmonary function variables in Caucasians. Of those, the glycoprotein gene network reached genome-wide significance for all four variables. P-value range p_meta_ = 6.29×10^−4^ - 2.80×10^−8^ on meta-analysis. In this study, we report on specific pathways that are significantly associated with pediatric lung function at genome-wide significance. In addition, we report the first loci associated with lung function in both pediatric Caucasian and African American populations**.**

## Introduction

The respiratory system plays a central role in overall health and lung-related disease. Spirometry is a commonly used technique to measure lung function as well as identify respiratory illness and follow lung disease progression. Commonly used spirometric indices include the forced expiratory volume in 1 second (FEV_1_), the forced vital capacity (FVC), and the FEV_1_/FVC ratio. Prior studies have demonstrated that pulmonary function can predict both pulmonary morbidity and mortality [1], [2].

Both genetic and environmental factors influence lung function. Population-based studies have described significant correlations between pulmonary function measures among monozygotic twins [3], and heritability estimates of lung function in excess of 40% have been reported [4], [5]. Longitudinal studies have also determined that the decline in lung function is also influenced by genetic factors [6]. While the heritability estimates of lung function are high, no definitive evidence of a single Mendelian gene with large effect underlying normal variation in lung function has been identified, indicating that a polygenic model of inheritance is most likely to determine lung function [5], [7]. In addition to a genetic determinants, environmental variables are known to affect lung function. Smoking is a well established environmental detriment to lung function, and children living near highways have also been shown to have significantly reduced lung function [8].

The first genome-wide association study (GWAS) to look at adult pulmonary function, specifically FEV_1_/FVC was reported in 2009 [9]. Recently, three large-cohort-based studies have reported a total of 28 additional loci significantly associated with pulmonary function measures in adults [10], [11], [12]. However, these loci are estimated to explain only a small portion of the variation in FEV_1_ and FEV_1_/FVC and do not address the unique aspects of pulmonary function in developing children or African-Americans. It is likely that a large number of loci influencing the heritable variation in lung function remain unidentified [10], [11], [12].

Two factors make children a unique population to study lung function. First, lung function rapidly increases from childhood to adolescence and by age 21 reaches a plateau phase followed by a slow but steady decline [13]. Thus, studying pulmonary function in children could yield associations with loci related to lung development which may not manifest phenotypic expression in adults. Second, it is possible that effects such as cigarette smoke exposure or other environmental factors over time may weaken phenotype expression and mask associations with genetic loci. It is plausible that the pediatric population may exhibit a stronger association between pulmonary function measures and genetic determinants.

Using pulmonary function data from children undergoing spirometry testing at the Children’s Hospital of Philadelphia, we conducted a GWAS to identify loci influencing pediatric lung function. To enhance study power, we additionally applied a pathway-based approach to search for gene networks influencing lung function. We and others have previously shown that GWAS derived gene network analyses can effectively uncover significant associations between gene pathways and complex diseases that are not discovered by individual SNP analyses [14].

## Materials and Methods

### Subjects

This study was approved by the institutional review board of the Children’s Hospital of Philadelphia. In accordance with IRB protocol, all subjects eligible for participation in this study were enrolled after obtaining informed written consent either from their parents, if under the age of 18 with the child’s assent, or directly from the subject if aged 18 or over. The study cohort consisted of children and young adults between 5–21 years of age that had completed pulmonary function testing and genotype analysis at the Children’s Hospital of Philadelphia. Only subjects of genetically inferred European ancestry or African ancestry were included in the study. Each ethnic cohort was analyzed separately to avoid issues with population stratification.

### Phenotype Data Collection

Lung function phenotype was defined using the percent predicted values for FVC, FEV_1_, FEV_1_/FVC, and forced expiratory flow between 25% and 75% of FVC (FEF_25–75%_) based on National Health and Nutrition Examination Study (NHANES III) reference values. Z-score was derived for each variable in each individual as follows, z-score = (actual value – predicted value)/standard deviation (SD). Calculation of the standard deviations was stratified by age, sex and ethnicity. The maximal Pre-bronchodilator z-score value was used in subsequent association analysis. Samples with values beyond 3SD from the mean were excluded from further analysis as outliers. Pulmonary function data from participating subjects was collected and stored using either the KoKo Spirometry System (Occupational Health Dynamics, Pelham AL) or the SpiroAir Pulmonary Function System (Morgan Scientific, Haverhill, MA). All data were transferred to a secure central databank for further analysis.

### Sample Collection and SNP Genotyping

Genomic DNA was collected from subjects’ whole blood, and all samples were genotyped on Illumina HumanHap550 BeadChip (Illumina, San Diego, CA) or the Illumina Human610-Quad version 1 BeadChip (Illumina, San Diego, CA), at the Center for Applied Genomics of The Children’s Hospital of Philadelphia. Illumina’s standard data normalization procedures and canonical genotype clustering files were used to process the genotyping signals and generate genotype calls.

### Quality Control

After genotyping, only samples with a call rate >95% and genotyping markers with a missing rate <5%, minor allele frequency >0.01 and HWE-p-value >0.0001 were retained for analysis. We further extracted markers shared by 550k and 610k chips for the association study. The population structure of all the samples was confirmed by Multi-Dimensional Scaling (MDS) analysis using PLINK software version 1.06 [15]. We detected cryptic related or duplicated samples in the cohort by determining the whole genome identity-by-descent (IBD) scores with PLINK software and excluded one sample from each pair with IBD scores >0.25.

### Association Analysis

Linear regression was performed at each SNP for the four lung function traits using PLINK software version 1.06 [15]. In the Caucasian cohort, a subset of the subjects had cystic fibrosis, asthma or cancer diagnoses, all of which were treated as covariates in the regression model. In the African American cohort, diagnoses of sickle cell disease, asthma and cancer were similarly treated as covariates. All four lung function phenotypes were approximately normally distributed (Figure S1), a linear regression model was utilized to evaluate the association between each of the phenotypes and the SNP genotype within Caucasian and African American cohorts separately. We also conducted meta-analyses using the PLINK and METAL packages [16].

### Genotype Imputation

We performed pre-phasing imputation of the two loci on chromosome 12 and 18 using SHAPEIT v2 [17] and IMPUTE2 [18], [19] with the 1000 Genome Phase I integrated variant set as reference panel (http://mathgen.stats.ox.ac.uk/impute/data_download_1000G_phase1_integrated.html). We further analyzed the imputed data using SNPTEST v2 [19] using an additive model in likelihood score test. SNPs with an imputation quality score <0.9 or minor allele frequency <0.01 were excluded from further analysis.

### Gene Network Analysis

For the gene network analysis all SNPs from the individual GWAs of each pulmonary function phenotype with a p-value <1.0×10^−3^ (Table S1 and Table S2) were mapped to their closest gene. This enrichment p-value threshold is similar and slightly more conservative than one used in a similar GWAS evaluating type II diabetes with follow-up pathway analyses in the Wellcome Trust Case Control Consortium (WTCCC) [20]. The resulting lung function gene lists for each of the four phenotypes (FEV_1_, FVC, FEV_1_/FVC and FEF_25–75%_), were then analyzed for functional annotation using the Database for Annotation, Visualization, and Integrated Discovery (DAVID) v6.7 program [21]. Results from the most significant pathway(s) for each cohort were meta-analyzed using a Z-transform method as implemented in the R package survcomp [22], weighted by the square-root of sample size of each cohort. Cochran’s *Q*-test for heterogeneity [23] was performed to check for substantial variation between studies stratified by asthma status. The top 2000 SNPs downloaded from the publication of Hancock et al. [10] and that of Repapi et al. [11] on genome-wide association studies of lung function were used as independent replication cohorts.

## Results

### Identification of SNPs Associated with Pulmonary Function Measures

We carried out a GWAS examining the relationship between SNP genotype data and pulmonary function results, including FEV_1_, FVC, FEV_1_/FVC and FEF_25–75%_. Individuals were genetically inferred as either Caucasian or African American based on the MDS analysis, and were evaluated separately in the GWAS analysis, followed by meta-analysis of the most significant loci and gene networks uncovered. The characteristics of each cohort are summarized in Table 1 and the distribution of the phenotype is shown in Figure S1.

**Table 1:** Study participant characteristics.

	Caucasian (%)	African American (%)
	**n = 1015**	**n = 541**
**Gender**		
Females	418 (41.18)	252 (46.58)
**Diagnosis**		
Cystic fibrosis	81 (7.98)	2 (0.37)
Sickle cell disease	2 (0.20)	63 (11.65)
Asthma	588 (57.93)	403 (74.49)
Cancer	65 (6.40)	8 (1.48)

After applying stringent quality control and filtering criteria on the genotyped SNPs, we analyzed 524k SNPs and 510k SNPs for our African American and Caucasian cohorts, respectively. Quantitle-quantile (Q-Q) plots (Figure S2) demonstrated no substantial deviation between observed and expected p-values genome-wide for any of the four pulmonary function measurements in each of the two cohorts. The Genomic Inflation Factor (GIF) for all GWA studies in the Caucasian and African American cohorts consistently ranged from 1 to 1.01, and 1.00 to 1.04, respectively, suggesting that there were no remarkable confounding factors in either cohort analysis.

While no SNP reached genome-wide significance in either cohort of our study, several loci showed trends towards association. In the Caucasian cohort, GWAS of FVC showed association of two SNPs at a locus on chromosome 18 with a p-value <1.0 ×10^−5^ (top SNP rs879500, p-value = 4.81**×**10^−6^), followed by multiple SNPs with a nominal p-value <0.05 (Figure S3). The SNPs map to an inter-genic region the closest neighboring gene to which is N-Cadherin protein (*CDH2*). Four SNPs at a second locus on chromosome 12 containing the myosin binding protein C (*MYBPC1*) gene also showed nominal association with FEV_1_ top SNP rs10860757, p-value = 6.67**×**10^−7^, with multiple other SNPs at p-values <0.05 (Figure S3). Imputation followed by association testing revealed additional 23 and 21 variants with p-value <1.0 ×10^−5^ in the above two loci respectively, though no SNP reached genome-wide significance likely due to the limited power of our study at the single SNP level. Both loci are worthy of further replication in cohorts of larger sample size.

There were also multiple SNPs that showed a trend towards association in the African American cohort. Two intronic SNPs in the catenin (cadherin-associated protein), alpha (CTNNA3) gene on Chromosome 10 had p-values <1.0 ×10^−5^ for FVC, and an additional 52 SNPs at this locus had p-values <0.05.

Subsequent meta-analysis of the association results for FEV1 from both Caucasian and African American cohorts revealed association at rs1982346 with a p-value of 1.41**×**10^−7^. The SNP maps to the *INTS12*/*GSTCD* region of chromosome 4, which has previously been shown to be associated with FEV_1_ in multiple studies, and has also been associated with FVC [11], [12].

### Functional Annotation Analysis

The number of genes meeting enrichment criteria for each lung function variable is listed in Table S1. Using the pediatric Caucasian group as a discovery cohort, functional annotation analysis of lung-function enriched gene lists using DAVID yielded several functional terms associated with FVC, FEV_1_, FEV_1_/FVC, and FEF_25–75%_. To account for multiple testing by DAVID, we used the Benjamini-Hochberg adjusted p-value [24] of <0.05 as criteria for evidence of genome-wide significance of association between analyzed gene sets and functional terms. The discovery cohort revealed 34 functional annotation terms related to lung function enriched gene groups forming functional networks. Specifically, FVC was significantly associated with 5 functional terms, FEV_1_ was significantly associated with 10 functional terms, and FEV_1_/FVC and FEF_25–75_ were associated with 15 and 4 terms, respectively (Table 2).

**Table 2:** Top functional annotation networks associated with lung function variables in Caucasian cohort.

Gene Network	Count	P-value	Benjamini-Hochberg CorrectedP-value
**FVC**			
GO: 0007155∼cell adhesion	31	2.80**×**10^−6^	0.00411
GO: 0022610∼biological adhesion	31	2.88**×**10^−6^	0.00212
cell adhesion	21	2.45**×**10^−5^	0.00755
PIRSF002504:cadherin	5	2.70**×**10^−4^	0.0345
*Glycoprotein*	101	9.13**×**10^−5^	0.00936
**FEV_1_**			
topological domain:Cytoplasmic	86	5.85**×**10^−5^	0.0337
topological domain:Extracellular	72	9.71**×**10^−5^	0.0225
Membrane	137	1.72**×**10^−4^	0.0173
*Glycoprotein*	99	5.64**×**10^−4^	0.0418
IPR003962:Fibronectin, type III subdomain	8	1.86**×**10^−6^	0.00111
IPR013098:Immunoglobulin I-set	12	3.61**×**10^−5^	0.0107
IPR003961:Fibronectin, type III	14	3.62**×**10^−5^	0.00716
domain:Fibronectin type-III 2	11	7.35**×**10^−5^	0.0283
domain:Fibronectin type-III 1	11	7.84**×**10^−5^	0.0227
SM00060:FN3	14	1.17**×**10^−4^	0.0165
**FEV_1_/FVC**			
*Glycoprotein*	111	4.39**×**10^−6^	5.17**×**10^−4^
glycosylation site:N-linked (GlcNAc…)	105	2.05**×**10^−5^	0.0126
topological domain:Extracellular	75	4.34**×**10^−5^	0.0177
topological domain:Cytoplasmic	86	1.62**×**10^−4^	0.0392
GO: 0007267∼cell-cell signaling	28	1.33**×**10^−5^	0.0213
ionic channel	22	1.74**×**10^−7^	6.13**×**10^−5^
GO: 0022836∼gated channel activity	22	4.85**×**10^−7^	2.13**×**10^−4^
GO: 0005216∼ion channel activity	24	1.29**×**10^−6^	2.83**×**10^−4^
GO: 0022838∼substrate specific channel activity	24	2.17**×**10^−6^	3.18**×**10^−4^
GO: 0015267∼channel activity	24	3.91**×**10^−6^	4.29**×**10^−4^
GO: 0022803∼passive transmembrane transporter activity	24	4.04**×**10^−6^	3.54**×**10^−4^
GO: 0005261∼cation channel activity	19	5.29**×**10^−6^	3.87**×**10^−4^
ion transport	27	9.16**×**10^−6^	8.08**×**10^−4^
GO: 0046873∼metal ion transmembrane transporter activity	20	1.66**×**10^−5^	0.00104
potassium channel	8	3.96**×**10^−4^	0.0173
**FEF_25–75%_**			
*Glycoprotein*	113	5.50**×**10^−8^	1.89**×**10^−5^
glycosylation site:N-linked (GlcNAc…)	109	8.31**×**10^−8^	9.92**×**10^−5^
topological domain:Cytoplasmic	86	1.70**×**10^−5^	0.0101
Membrane	135	9.37**×**10^−5^	0.0107

### Functional Annotation Analysis Replication Testing

Only gene networks that exceeded a Benjamini-Hochberg corrected p-value <0.05 in the discovery cohort based on the functional annotation terms applied for each of the lung function variables were selected for replication. Although FVC, FEV_1_, FEV_1_/FVC and FEF_25–75%_ have the ability to measure discrete aspects of lung function, the four variables have an inherent degree of interrelatedness. Thus, we would expect that only functional terms truly related to lung function would appear as a candidate term in all four lung function variables. Therefore, to detect functional terms truly associated with lung function measures during replication testing, and to apply an additional level of *a priori* stringency to the candidate selection process, we limited the replication process to functional terms common to all four studied pulmonary function measures. Evaluation of 34 candidate terms from DAVID revealed that only the glycoprotein term satisfied these criteria.

All multiple-testing corrected p-values for the glycoprotein functional annotation network during replication testing are listed in Table 3. In the African American pediatric cohort, we observed significant associations (Benjamini-Hochberg corrected p-values <0.05) between the glycoprotein term and FVC, FEV_1_, FEV_1_/FVC and FEF_25–75%_. In a separate validation step, we used existing data from two separate published GWA studies identifying significant associations between novel loci and lung function measures in adults. Using the top 2,000 published SNPs and closest genes associated with either FEV_1_ (maximum p-value = 1.35**×**10^−4^) or FEV_1_/FVC (maximum p-value = 4.30**×**10^−4^) from a large scale GWA of the Cohorts for Heart and Aging Research in Genomic Epidemiology (CHARGE) Consortium [10], we pursued validation testing in DAVID. Briefly, the CHARGE consortium is a compendium of smaller cohorts totaling 21,109 adults of European ancestry. The mean age of the cohorts comprising the CHARGE consortium ranged from 54.3 to 74.5 years of age [10]. Based on DAVID analysis, we observed significant associations between lung function enriched gene sets and the glycoprotein functional term for FEV_1_ (p-value = 1.91**×**10^−5^) and FEV_1_/FVC (p-value = 6.42**×**10^−4^). Furthermore, we used the top 2000 SNPs associated with lung function measures FEV_1_ and FEV_1_/FVC from a GWAS of 20,288 individuals of European ancestry in the SpiroMeta consortium [11] as a replication cohort to test our findings. The age range of SpiroMeta participants was from 8 to 91 years [11]. In order to generate lung function enriched gene lists for FEV_1_ and FEV_1_/FVC, we used the SNP-Nexus program to map all SNPs to the closest gene [25]. DAVID analysis of both gene sets yielded significant associations (Benjamini-Hochberg corrected p-values <0.05) between FEV_1_ (p-value = 6.65**×**10^−3^) and FEV_1_/FVC (p-value = 1.57**×**10^−2^) and the glycoprotein functional term. Finally, we did meta-analysis of the four cohorts for glycoprotein functional term, which yielded a result with p-value <0.001 for each variable (p-value = 6.29**×**10^−4^ for FVC, p-value = 1.40**×**10^−7^ for FEV_1_, p-value = 1.15**×**10^−6^ for FEV_1_/FVC and p-value = 2.80**×**10^−8^ for FEF_25–75%_) (Table 3). The observed association between the glycoprotein functional term and FEV1 and FEV1/FVC were therefore replicated in two adult population studies.

**Table 3:** Functional analysis results: Benjamini-Hochberg corrected p-values for glycoprotein term association with lung function variables in pediatric and adult cohorts.

Variable	Caucasian Cohort	African American Cohort	CHARGE Cohort	SpiroMeta Cohort	Meta-analysis
FVC	9.36**×**10^−3^	1.22**×**10^−2^	–	–	6.29**×**10^−4^
FEV_1_	4.18**×**10^−2^	5.50**×**10^−3^	1.91**×**10^−5^	6.65**×**10^−3^	1.40**×**10^−7^
FEV_1_/FVC	5.17**×**10^−4^	6.34**×**10^−6^	6.42**×**10^−4^	1.57**×**10^−2^	1.15**×**10^−6^
FEF_25–75%_	1.89**×**10^−5^	1.81**×**10^−4^	–	–	2.80**×**10^−8^

### Stratified Functional Annotation Analysis

As a considerable proportion of the children in our cohorts suffered from respiratory disease, most notably asthma, we carried further analyses to ensure that glycoprotein pathway association was not confounded by the disease status. We carried out stratified pathway analyses on asthma status even though the SNP association analyses had been adjusted for presence of disease terms by including them as covariates. In the Caucasian cohort, there are 285 children without respiratory disease diagnosis and 588 children with asthma. We conducted a GWAS analysis in the above two subgroups of children separately followed by the functional annotation analysis using DAVID. In the African American cohort, the number of respiratory disease free individuals was too small (n = 75) to warrant subgroup analysis. We examined the glycoprotein term in the functional annotation analysis for each pulmonary function variable. As shown in Table 4, the p-values for glycoprotein term are <0.05 for each pulmonary function variable in each subgroup. We further examined whether large variation in the significance of glycoprotein pathway was present between subgroups. The p-value of homogeneity test for each lung function variable within the Caucasian cohort was >0.1, which did not indicate any between-study heterogeneity in the Caucasian cohort. The results suggest that the significant association between functional term glycoprotein and pulmonary measures is independent of asthma status.

**Table 4:** Stratified functional analysis results: nominal p-values for glycoprotein term association with lung function variables in the Caucasian pediatric cohort.

variable	Caucasian				
	healthy (n = 285)	asthma (n = 588)		Combined (n = 873)	
	P-value	P-value	HetPval^a^	P-value	Benjamini-Hochberg Corrected P-value
FVC	1.69**×**10^−4^	1.78**×**10^−6^	0.721	1.10**×**10^−4^	0.0165
FEV1	1.43**×**10^−4^	1.45**×**10^−4^	0.342	4.14**×**10^−7^	1.19**×**10^−4^
FEV1/FVC	8.24**×**10^−5^	3.49**×**10^−4^	0.235	2.13**×**10^−4^	0.0157
FEF_25–75%_	1.24**×**10^−3^	8.90**×**10^−6^	0.911	3.99**×**10^−5^	0.0123

a test for heterogeneity p-value.

We also conducted meta-analysis of the healthy and the asthma subgroups in the Caucasian cohort at the SNP level followed by functional annotation analysis. At the SNP level, we observed the similar trend of association between the 18q12.1 locus and FVC, as well as that between MYBPC1 and FEV1. The results of functional annotation analysis for glycoprotein term are shown in Table 4 column “combined (n = 873)”. The glycoprotein term remained genome-wide significant with Benjamini-Hochberg corrected p-value <0.05 for each pulmonary function measure.

## Discussion

In a GWAS of 1015 children of European ancestry, we found that the class of glycoprotein genes based on functional annotation terms in DAVID was significantly associated with all evaluated pulmonary function measures. Replication analysis in a cohort of African American children and in two independent primarily adult cohorts confirmed the association of the glycoprotein gene network and both FEV_1_ and FEV_1_/FVC.

Pulmonary function testing is a vital component of understanding the physiological properties of the lung, and recent efforts to identify the genetic determinants have demonstrated multiple loci affecting lung function. While GWAS is a vital tool in ascertaining gene-phenotype associations, the polymorphisms identified using this method alone to date have uncovered a relatively small percentage of the estimated heritability in complex traits and diseases, and only a limited number of loci have been identified. Because conservative stringency levels are required to avoid spurious associations and detect true causal variants, pathway and functional analyses may be useful in uncovering variants missing GWAS significance thresholds in single test analyses.

In our population, GWAS detected SNPs that are of p-value <10^−5^ in both the Caucasian and African American cohorts. In the Caucasian cohort, rs879500 and rs1566819, two SNPs near the N-cadherin gene (*CDH2*), were associated with FVC (p-value = 4.81**×**10^−6^ and p-value = 6.52**×**10^−6,^ respectively). *CDH2* encodes for a calcium dependent transmembrane glycoprotein involved in cell-cell adhesion essential to tissue integrity [26]. *CDH2* was originally discovered in lung mesenchymal cells, and is expressed in bronchial epithelial cells during weeks 12–40 of early human lung development, with less expression in the adult lung [27]. Increased expression of *CDH2* in mesenchymal cells has been hypothesized to play a role in idiopathic pulmonary fibrosis [28]. Additionally, *CDH2* is strongly expressed in neuroendocrine cell lung tumors [29]. Four SNPs, rs10860757, rs6538998, rs7308665, and rs12146808 were associated with FEV_1_ (p-value = 6.67**×**10^−7^, p-value = 1.26**×**10^−6^, p-value = 1.48**×**10^−6^, and p-value 4.46**×**10^−6^ respectively) and found to reside in the intronic regions of the myosin binding protein C1 (*MYBPC1*) gene. *MYBPC1* is a myosin-associated slow skeletal muscle isoform that is found in the C-region of A bands in striated muscle. The significance of *MYBPC1* has not been fully determined, though mutations in this gene are associated with type 1 distal arthrogryposis [30]. The diaphragm and intercostal muscles, the primary muscles of respiration, are predominantly composed of slow skeletal muscle fiber, and play a vital role in lung function mechanics. Thus, the SNP signals related to the *MYBPC1* gene may be related to respiratory skeletal muscle function.

In the African American cohort, two SNPs (rs1471384, p-value = 3.89**×**10^−6^ and rs4746554, p-value = 3.95**×**10^−6^) in the intron of catenin (cadherin associated protein) alpha 3 (*CTNNA3*) on chromosome 10 were found to be associated with FVC. The *CTNNA3* protein is proposed to play a role in cell-cell adhesion [31]. recently a GWAS identified *CTNNA3* as a susceptibility gene for airway hyperresponsiveness in toluene diisocyanate induced asthma [31], and mutations in *CTTNA3* have been implicated in NSCL cancer [32].

While our study did not uncover any single variant association at a genome-wide level, it did reveal associations between glycoprotein genes as a class and lung function measures based on functional annotation of the glycoprotein gene network in DAVID. Since the process of pulmonary function evaluation involves testing airflow through airways, the resistance, R, to air flow in the airways can be described by Pouiselle’s law, with *r* representing the relationship of airway radius to the resistance to airflow:
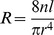



Thus, factors that slightly alter the dimensions of the airway radius will have a large inverse effect on lung function. This effect is most commonly seen in the extreme form in obstructive disease processes where reduced airway diameter due to airway occlusion may result in substantially reduced lung function.

While glycoproteins are ubiquitous in nature, their contributions to complex inter- and intra-cellular processes are less well known. In the respiratory tract, glycoproteins are critical participants in both regulatory and pathologic processes. The airways of the healthy lung are coated with a mucous layer rich in glycoproteins, that protects airway epithelium from harmful environmental agents and pathogenic organisms. Glycoproteins may have variable effects on the airway mucous layer of large and small airway radius, potentially resulting in variations among individuals with normal lung function.

Airway diameter, and therefore lung function, may also be determined during early respiratory system development. Several key glycoproteins have already been implicated in lung development and branching morphogenesis in early mammalian organogenesis [33], [34], [35]. In post-natal lung development, both mechanical and environmental factors are thought to play a prominent role in continued lung growth. Through breathing effort, movement of the chest wall produces a potent growth stimulus to lung tissue stimulating a cascade of cellular signaling resulting in gene expression that drives lung growth. Cytoskeletal glycoproteins and cell-cell adhesion molecules have been implicated in this pathway [36]. Additionally, complex environmental-genetic interactions are thought to influence final adult lung function [8]. Given that our entire population falls within the age range encompassing dynamic post-natal lung growth, it is possible that our gene sets may consist of glycoprotein-related factors that are determinants in both early and late lung development and function.

Our study has several important strengths. While prior investigators have identified lung function genes in large adult studies with a wide range of ages, this study focused on the pediatric population. By limiting our study population to a relatively smaller age range (5–21 years), our findings may include genes that are associated with lung function development. Environmental effects are well described as negatively influencing lung function, chief among these factors being exposure to cigarette smoke and ever-smoker history [8]. While individuals at any age may become smokers or live in homes with second hand smoke exposure, overall it is less likely that our population’s lung function is influenced by this factor, or the lifetime accumulation of other detrimental chronic exposures.

Most importantly, our GWAS was the first study to attempt to identify lung function loci in a cohort of children exclusively of African American ancestry. Our method did identify gene sets with significant associations to lung function in this cohort, and are the first genes to be reported to be related to lung function in individuals of African American ancestry. In a recent study, Kumar and colleagues determined that increasing percentage of African American ancestry is inversely related to FEV_1_, suggesting the possibility of misclassification of disease severity when using race-based normative data [37]. Identification of polymorphisms and loci specific to this population that affect lung function may begin to explain the inherent discrepancy between normative values and African American ancestry.

The relatively high proportion of asthmatic cases within each cohort raised the possibility that the results of the pathway analysis may be confounded by the respiratory disease process. To address this possibility we carried out stratified analyses on the respiratory health status. Statistical analyses in the Caucasian cohort did not indicate substantial heterogeneity between subgroups. Furthermore, the association of glycoprotein with FEV1 and FEV1/FVC were replicated in the published adults cohorts in which samples were from the general population of European Ancestry. Therefore, the important role of glycoprotein gene network in lung function is unlikely to be limited to asthmatic conditions.

We have provided a resource of our top SNPs from our GWAS, as well as the lung-function specific gene sets from our network analysis for each lung function phenotype to facilitate future analyses. More detailed investigation into these gene sets may provide more insight into the molecular mechanisms governing lung function in children, and uncover potential targets for pharmacotherapy in respiratory-related diseases.

## Supporting Information

Figure S1 **Histograms of pulmonary function measures among Caucasian and African American Children.**
(JPG)

Figure S2 **The Q-Q plot for each GWAS in Caucasian and African American Children.**
(JPG)

Figure S3 **Manhattan plots for association testing of pulmonary function measures FVC and FEV1 in Caucasian children.**
(JPG)

Table S1 **Number of genes from GWAS results used for functional analysis of lung function phenotypes.**
(DOC)

Table S2 **SNPs with p-value <1.0×10^−3^ from GWAS results for each pulmonary function measure in Caucasian and African American cohorts.**
(XLS)
