# Supplementary figures and images for: Gene Network Analysis in a Pediatric Cohort Identifies Novel Lung Function Genes
Source: PLoS One. 2013 Sep 2;8(9):e72899. doi: 10.1371/journal.pone.0072899 (PMC3759429; doi:10.1371/journal.pone.0072899)

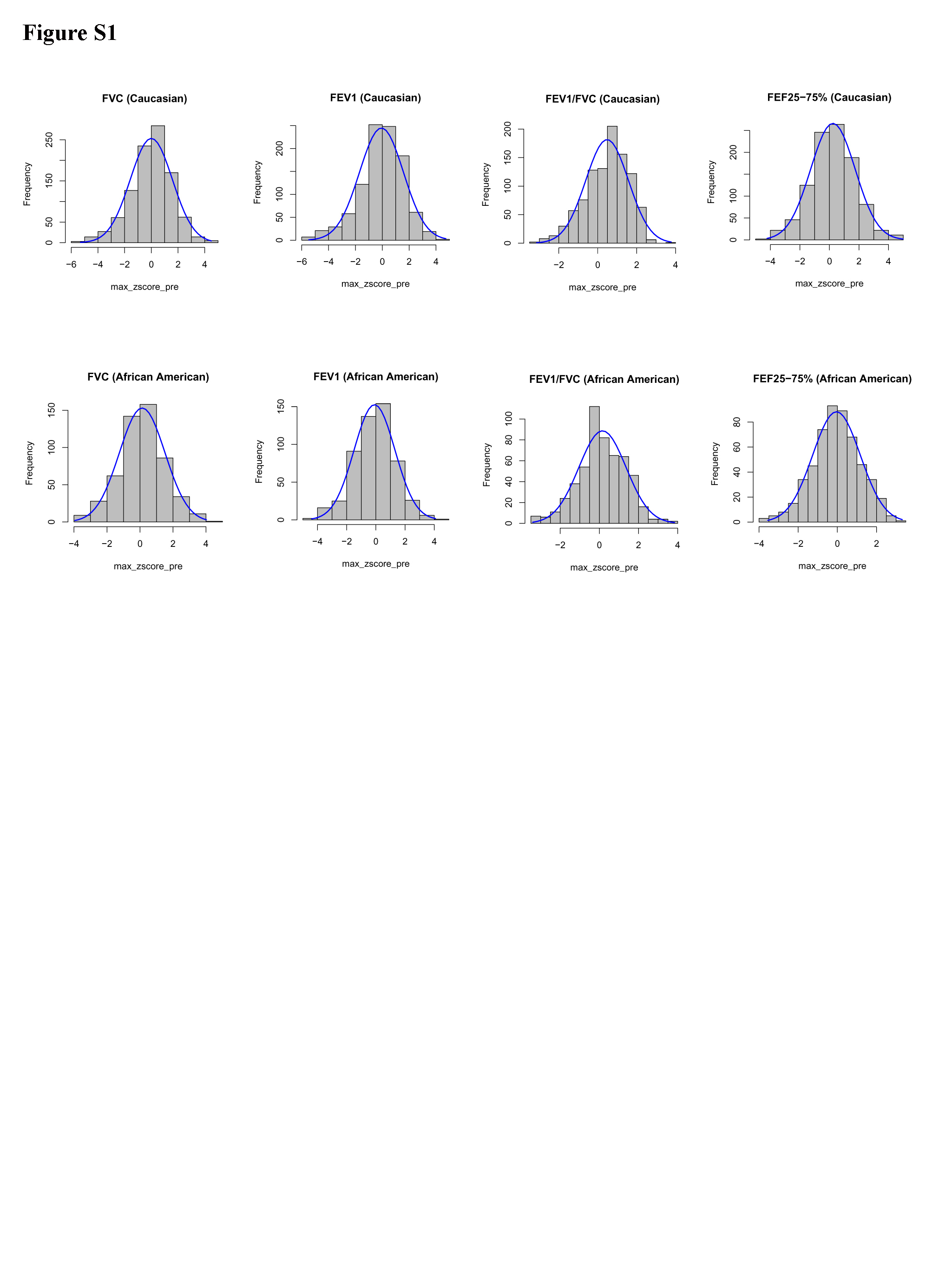

Supplement: Figure S1 — Histograms of pulmonary function measures among Caucasian and African American Children. (JPG) [file pone.0072899.s001.jpg]

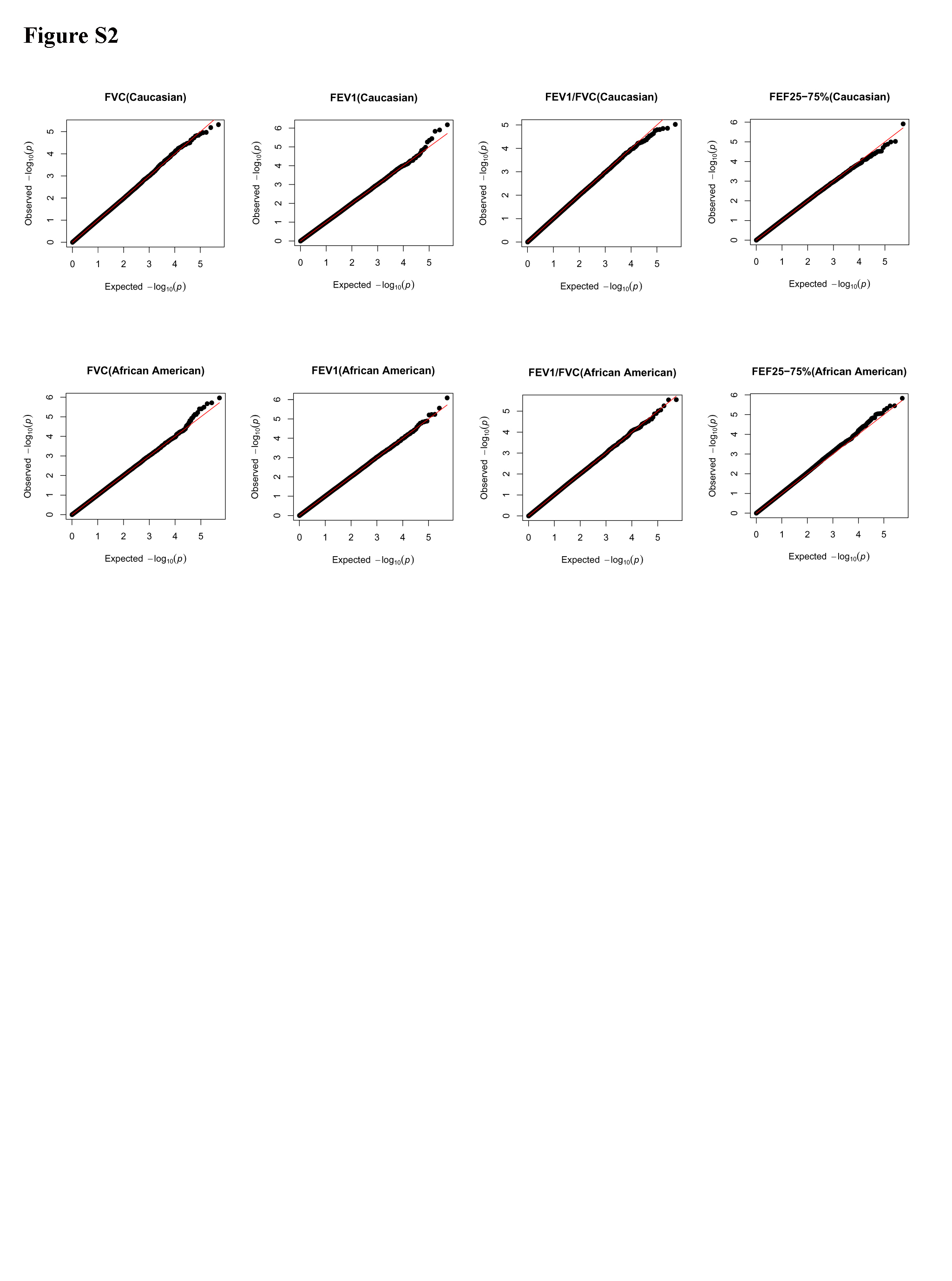

Supplement: Figure S2 — The Q-Q plot for each GWAS in Caucasian and African American Children. (JPG) [file pone.0072899.s002.jpg]

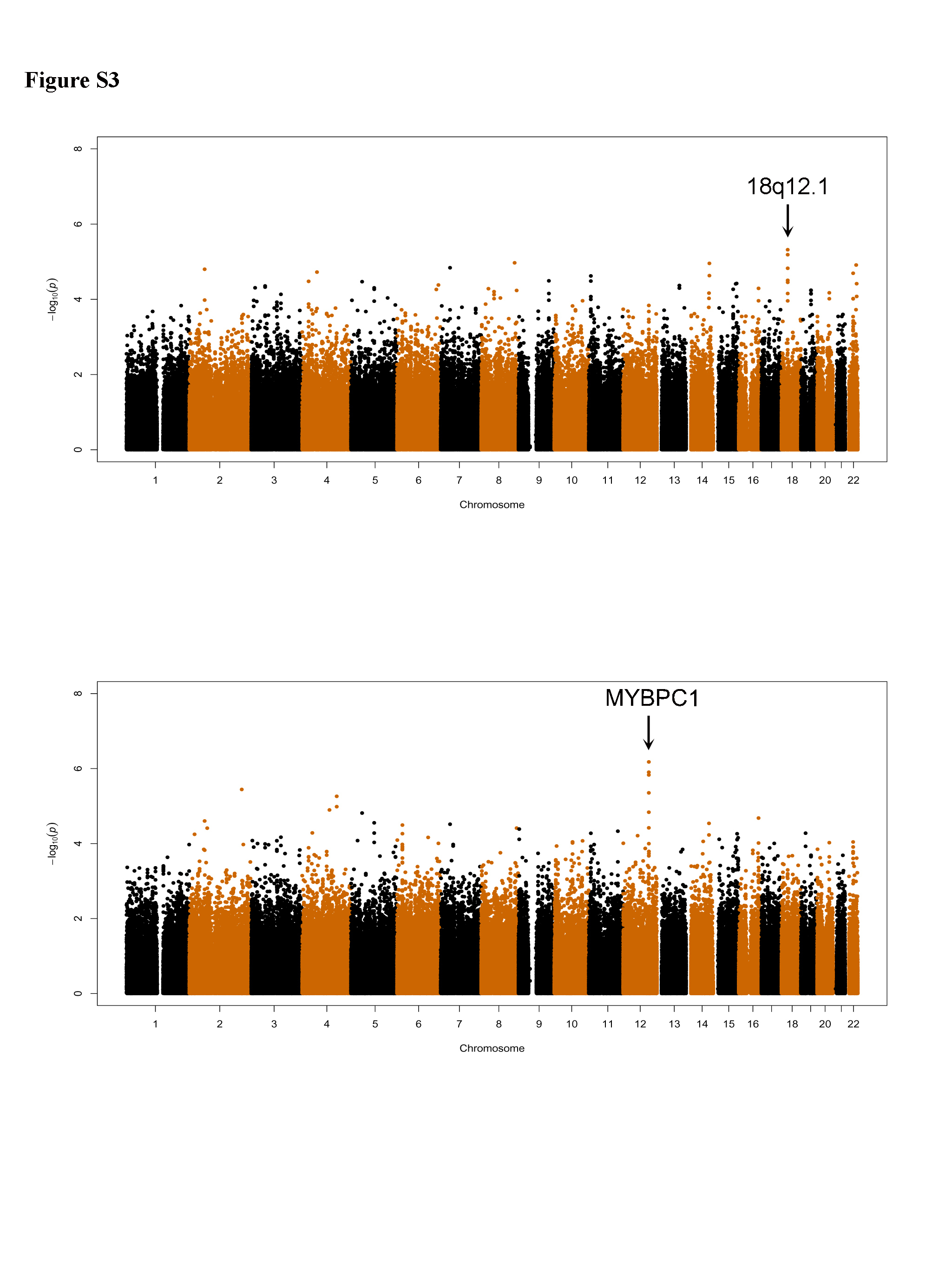

Supplement: Figure S3 — Manhattan plots for association testing of pulmonary function measures FVC and FEV1 in Caucasian children. (JPG) [file pone.0072899.s003.jpg]
