# Supplementary material for: Gene Network Analysis in a Pediatric Cohort Identifies Novel Lung Function Genes
Source: PLoS One. 2013 Sep 2;8(9):e72899. doi: 10.1371/journal.pone.0072899 (PMC3759429; doi:10.1371/journal.pone.0072899)
Supplement: Table S1 — Number of genes from GWAS results used for functional analysis of lung function phenotypes. (DOC) [file pone.0072899.s004.doc]

**Table S1. Number of genes from GWAS results used for functional analysis of lung function phenotypes.**

|  | **Genes with p-value < 1.0 x 10^-3^**  **(% GWAS results)** |
| --- | --- |
| **FVC** |  |
| Caucasian | 329 (1.9) |
| AA | 411 (2.4) |
| **FEV_1_** |  |
| Caucasian | 331 (1.9) |
| AA | 430 (2.5) |
| **FEV_1_/FVC** |  |
| Caucasian | 355 (2.1) |
| AA | 402 (2.3) |
| **FEF_25-75%_** |  |
| Caucasian | 330 (1.9) |
| AA | 508 (3.0) |
